# Omega-6/omega-3 fatty acid intake of children and older adults in the U.S.: dietary intake in comparison to current dietary recommendations and the Healthy Eating Index

**DOI:** 10.1186/s12944-018-0693-9

**Published:** 2018-03-09

**Authors:** Kelly W. Sheppard, Carol L. Cheatham

**Affiliations:** 1 Center for Biobehavioral Health, The Research Institute at Nationwide Children’s Hospital, 700 Children’s Drive, Columbus, OH 43205 USA; 2 Department of Psychology & Neuroscience, University of North Carolina at Chapel Hill, 235 E. Cameron Ave, Chapel Hill, NC 27514 USA; 3 Nutrition Research Institute, University of North Carolina at Chapel Hill, 500 Laureate Way Rm 1101, Kannapolis, NC 28081 USA

**Keywords:** Docosahexaenoic acid, Typical diet, Fatty acid intake, Omega-6/omega-3 fatty acid ratio, Human children, Human older adult

## Abstract

**Background:**

Omega-6 and omega-3 fatty acids (FAs) and their ratio have been shown to affect cognitive function in children and older adults. With these analyses, we aimed to describe omega-6 and omega-3 FA intake among children and older adults in light of FA intake recommendations and with consideration of overall diet.

**Methods:**

Data were merged from two cross-sectional studies with 219 children 7 to 12 years old and one longitudinal study with 133 adults 65 to 79 years old. Demographic data, anthropometric data, and Healthy Eating Index scores were used to study relations among the omega-6 to omega-3 FA ratio and age, education, body mass index, and diet quality. FA intake, demographic, and anthropometric data were examined using partial correlations, t-tests, and analysis of variance.

**Results:**

Most children and adults consumed at least the recommended amount of alpha-linolenic acid (LNA; omega-3) for their age and gender without consuming high amounts of linoleic acid (LA; omega-6), but did not consume sufficient eicosapentaenoic acid (EPA; omega-) and docosahexaenoic acid (DHA; omega-3). The average omega-6 to omega-3 ratios in both groups were lower than previously reported. Eating lower ratios was associated with healthier diets and consuming adequate amounts of several other nutrients. No demographic or anthropometric variables were related to FA intake in children. Adults with a college degree had significantly lower ratios than those without a college degree.

**Conclusions:**

American children and older adults are able to consume more balanced omega-6 to omega-3 ratios than has been indicated by commodity data. However, very few American children met even the lowest recommendations for EPA and DHA intake. Research is needed to clarify recommendations for the optimal ratio across development, which may aid in increasing EPA and DHA intake and improving health outcomes in the United States.

**Trial registration:**

ClinicalTrials.gov NCT02199808 13 July 2014, NCT01823419 (retrospectively registered) 20 March 2013, and NCT01515098 18 January 2012.

## Background

It is important to understand fatty acid consumption as part of the overall diet to align recommendations and scientific research with actual practice. Much has been made of the imbalanced consumption of fatty acids over recent decades [1–4] due to the demonstrated importance of fatty acids to the brain [5–8] and cognitive function [9–12]. Data from food commodities have indicated increasing imbalance in the n-6/n-3 ratio in the food supply over time [2, 13], with some reviews indicating ratios above 20:1 [1, 14, 15]. However, smaller studies using intake from 24-h diet recalls or records, and even the National Health and Nutrition Examination Survey (NHANES) data, often find ratios closer to 10:1 [16–19]. Specifically, the increase in n-6 coupled with the decrease in n-3 in the food supply has produced speculation that most people consume well above recommended amounts of n-6 and well below recommended amounts of n-3 [1, 2, 14]. In practice, people often manage to consume much closer to recommendations on average, despite alterations in the overall food supply [18, 20].

One issue that is still unclear is what range of n-6/n-3 ratios would be optimal, and how the optimal ratio will change with development. Papers discussing likely ancestral diets have pointed to 1:1 as a goal [14, 21], and yet it is unclear how accurate those reports can be and if mimicking the ancestral diet is even desirable. Despite claims that the human genome has not changed much in the last 10,000 years [14, 22], recent advances in genetics and epigenetics would suggest that the present day environment can have a powerful impact on nutrient requirements [23–25] through slight differences in gene function created by single nucleotide polymorphisms (SNPs) and changes in gene expression through methylation, acetylation, and histone modification.

A second issue is that it is important to investigate optimal fatty acid intake from a developmental perspective as recommendations should likely differ in response to specific needs. Periods of rapid brain growth will require different nutritional support compared to periods of synaptogenesis, skill consolidation, or re-organization. Cognitive functions supported by the frontal cortex and the hippocampus should be a focus due to the accumulation of n-6 and n-3 fatty acids in those brain areas [26, 27] and evidence from animal models [5–8]. With the present analyses, we aimed to characterize fatty acid intake among children 7 to 12 years old, when abilities subserved by the hippocampus and frontal cortex steadily improve, and older adults 65 to 79 years old, when abilities subserved by the hippocampus and frontal cortex steadily decline.

Current recommendations for n-6 and n-3 fatty acid intake do not align with evidence from research on the importance of the balance of n-6 and n-3 intake [20]. There are no current guidelines for the n-6/n-3 ratio, but the current recommendations for n-6 and n-3 intake can be used to calculate what dietary n-6/n-3 ratio a person would have if they followed recommendations. For instance, current recommendations for children between 4 and 8 years old are to consume 10 g of linoleic acid (LA, n-6) and 0.9 g of alpha-linolenic acid (LNA, n-3) combined with either 0.1 g or 0.2 g of docosahexaenoic acid (DHA, n-3) and eicospentaenoic acid (EPA, n-3), producing an n-6/n-3 ratio of 9.1–10.1 [28]. Similarly, recommendations for males and females between 9 and 13 years old range from ratios of 7.1 (9 to 13 year old males) to 10.9 (9 to 13 year old females). Even if ratios of 1:1 are not the goal at every age, researchers have generally found ratios below 10 to be more optimal [29, 30]. Additionally, there isn’t any evidence that there should be such a large difference in the balance consumed by males and females 9 to 13 years old. If anything, recent evidence points to lower ratios during puberty (for both sexes) to support brain changes occurring during that time [9].

Data were merged from two cross-sectional studies that included children 7 to 12 years old [9, 29] and one longitudinal study of adults 65 to 79 years old (unpublished data). Analyses were conducted to characterize typical dietary intake of fatty acids and the ratio of n-6 to n-3 fatty acids. In addition, we sought to describe fatty acid intake in relation to the diet as a whole. Thus, the intake of n-6 and n-3 fatty acids were compared to the intake of other nutrients and to the calculated Healthy Eating Index (HEI) scores, which are a measure of the overall healthfulness of the diet. Differences in dietary n-6/n-3 ratios based on demographic and anthropometric characteristics were also explored.

## Methods

### Participants

Data from three studies previously conducted were merged. Criteria for inclusion required that the participants had provided at least three days of dietary recall data in the previous studies, whether they had completed the parent studies or not. The resulting data set included children and older adults, the specifics of which are detailed next. The three studies had the approval of the University of North Carolina at Chapel Hill’s Institutional Review Board, and the participants either gave informed consent (adults and parents) or informed assent (children) as required by the Declaration of Helsinki.

#### Children

Two hundred nineteen children 7 to 12 years old who participated in two studies on the role of fatty acids in executive function task performance from October 2010 to August 2015 were included in these analyses. From the first study [29], 70 were included in these analyses. From the second study [9], 149 were included. In the second study, only 78 children were brought to the lab to complete cognitive tests, and thus, height, weight, and other identifying data are only available on this subset. Table 1 shows the demographic characteristics of the 7- to 12-year-olds, and more details about the full studies can be found in the primary outcome papers [9, 29].

**Table 1:** Demographic and anthropometric data for 219 7 to 12 year old children and 133 65 to 79 year old adults

7- to 12-year-olds		65- to 79-year-olds	
Age (years)	8.8 (1.6)	Age (years)	72.1 (4.3)
7- to 9-year-olds	72.6% (159)	65- to 69-year olds	32.0% (41)
10- to 12-year-olds	27.4% (60)	70- to 74-year-olds	35.9% (46)
		75- to 79-year olds	32.0% (41)
Race		Race	
Caucasian	86.4% (140)	Caucasian	96.9% (124)
African-American	6.8% (11)	African-American	1.6% (2)
Asian	1.2% (2)	Asian	0.8% (1)
Native American	0.6% (1)	Native American	0.8% (1)
Multiracial	4.9% (8)	Multiracial	0%
Hispanic	5.6% (9)	Hispanic	0%
Female	53.0% (116)	Female	58.6% (75)
Maternal Education		Education	
High School Degree	5.6% (9)	Less than High School	2.3% (3)
Some College	31.7% (51)	High School Degree	14.1% (18)
College Degree	35.4% (57)	Some College/AA Degree	47.7% (61)
Graduate School	1.2% (2)	College Degree	19.5% (25)
Graduate Degree	26.1% (42)	Graduate School	14.8% (19)
		Graduate Degree	1.6% (2)
BMI	19.1 (5)	BMI	27.3 (3.8)
		Blood pressure	131.7 (17.3)/ 74.3 (9.1)
		Waist Circumference	39.5 (3.9)
		Mild Cognitive Impairment ^a^	66.4% (85)

*BMI* Body Mass Index, *AA* Associate of Arts

Note ^a^Mild cognitive impairment was assessed with the Montreal Cognitive Assessment (MoCA). Scores below 25 (out of 30) indicated mild cognitive impairment

#### Older adults

One hundred thirty-three adults 65 to 79 years old who participated in a larger study on the effect of blueberries on cognitive decline from February 2012 to May 2015 were included in these analyses. The 133 participants included in these analyses participated in at least the first session. Table 1 shows the demographic characteristics of the 65- to 79-year-olds, and more details on the full study can be found on ClinicalTrials.gov (NCT01515098).

### Dietary assessment with 24-h diet recalls

In all cases, data were collected on two weekdays and one weekend day to capture the variety of dining circumstances that occur within a typical week. Each child participant along with her or his caregiver completed three record-assisted 24-h diet recalls to obtain a snapshot of their typical diet following recommendations as described previously [31]. Older adults completed three 24-h dietary records that they brought to the lab to review with a researcher. Dietary records are considered more accurate with older adults because of the typical decline in memory with age [32]. These methods are generally considered the best methods for obtaining accurate dietary information from the respective age groups [31, 33]. Data collection was guided by and entered into the Nutrition Data System for Research (NDSR) developed by the Nutrition Coordinating Center, University of Minnesota, Minneapolis, MN. Additionally, most participants (61%) provided weight and height at an in-person visit to the lab using the same scale and stadiometer, and the older adults also provided waist circumference and blood pressure. The participant or the participant’s caregiver completed a demographic questionnaire at the lab visit.

### Estimation of n-6 and n-3 fatty acid intake

The NDSR database includes the content of a large number of nutrients (including n-6 and n-3 fatty acids) for each food entered, and uses those estimates to generate daily consumption values. To estimate typical intake, daily intake data from the three diet recalls were averaged to calculate daily nutrient totals and daily servings of foods. The n-6/n-3 ratio was calculated by dividing the average daily intake of all n-6 fatty acids by the average daily intake of all n-3 fatty acids.

### Calculation of HEI scores

HEI scores were calculated using the instructions provided in the NDSR manual (Nutrition Coordinating Center, Minneapolis, MN), which had been updated to include the 2013 changes to the HEI-2010 [34]. Briefly, the HEI score is comprised of 12 categories thought to be key to measuring the healthfulness of the diet, including intake of fruits and vegetables, whole and refined grains, protein and seafood, sodium, dairy, fatty acids, and empty calories (defined as solid fats, alcohol, and added sugars). The food servings are transformed to per 1000 kcal of overall energy intake, making the scores comparable across age groups. Quality scores are then assigned for each of the 12 categories based on proximity to recommendations, and the quality scores are totaled to create an HEI that can range from 0 to 100, with higher HEI scores indicating a healthier diet. Dietary adequacy was also assessed by comparing the participant’s nutrient intake with the recommendations (recommended daily allowance (RDA) or adequate intake (AI)) for their age and gender. Those who consumed at least 100% of their recommended intake received a 1, and those who consumed less than 100% received a 0. Means, standard deviations, and percent adequate intake for the nutrients and HEI scores are shown in Table 2.

**Table 2:** Nutrient intake and HEI scores in 219 7- to 12-year-old children and 133 65- to 79-year-old adults

7- to 12-year-olds	Mean Intake (SD)	Recommended Intake	% Adequate
Energy (kcal)	1845.6 (439.6)	Inactive: 1400 Moderately active: 2000 Highly active: 2400	
LNA (g)	1.4 (0.6)	4–8 yo: 0.9 F 9–13 yo: 1 M 9–13 yo: 1.2	71.8%
LA (g)	12.6 (5.0)	4–8 yo: 10 F 9–13 yo: 12 M 9–13 yo: 10	61.4%
DHA + EPA^a^	0.1 (0.3)	FAO [35]: 0.2	6.8%
		IOM [28]: 10% of LNA recommendation	14.1%
N-6/n-3 ratio	9.2 (2.8)		
PUFA (% of calories)	6.8 (1.9)	All 6–11%	60%^b^
MUFA (% of calories)	11.2 (2.5)		
SFA (% of calories)	11.1 (2.1)	All < 10%	31.4%
Total Fiber (g)	14.3 (5.4)	4–8 yo: 25 F 9–13 yo: 26 M 9–13 yo: 31	3.6%
Vitamin A (mcg)	824.9 (510.1)	4–8 yo: 400 9–13 yo: 600	72.3%
Vitamin B6 (mg)	2.0 (1.8)	4–8 yo: 0.6 9–13 yo: 1	94.1%
Vitamin B12 (mcg)	6.0 (7.8)	4–8 yo: 1.2 9–13 yo: 1.8	95.5%
Vitamin C (mg)	94.0 (114.0)	4–8 yo: 25 9–13 yo: 45	81.8%
Vitamin D (mcg)	7.0 (5.4)	All: 5	72.3%
Vitamin E (mg)	7.2 (3.5)	4–8 yo: 7 9–13 yo: 11	26.8%
Vitamin K (mcg)	78.5 (112.4)	4–8 yo: 55 9–13 yo: 60	46.4%
Calcium (mg)	965.1 (386.3)	4–8 yo: 800 9–13 yo: 1300	38.6%
Iron (mg)	15.4 (7.6)	4–8 yo: 10 9–13 yo: 8	85.9%
Sodium (mg)	2861.0 (830.7)	4–8 yo: 1200 9–13 yo: 1500	98.2%
Potassium (mg)	1990.0 (684.0)	4–8 yo: 3800 9–13 yo: 4500	1.4%
Zinc (mg)	10.6 (4.8)	4–8 yo: 5 9–13 yo: 8	82.7%
Pantothenic Acid (mg)	5.3 (3.8)	4–8 yo: 3 9–13 yo: 4	66.4%
Choline (mg)	247.8 (100.6)	4–8 yo: 250 9–13 yo: 375	25.5%
Folate (mcg)	382.6 (179.9)	4–8 yo: 200 9–13 yo: 300	80.9%
Water (g)	1585.8 (638.5)	4–8 yo: 1700 F 9–13 yo: 2100 M 9–13 yo: 2400	19.6%
HEI	53.3 (12.6)		
65- to 79-year-olds	Mean Intake (SD)	Recommended Intake	% Adequate
Energy (kcal)	1780.3 (479.0)	Inactive: 2000 Moderately Active: 2400 Highly Active: 2600	
LNA (g)	1.6 (0.8)	Males: 1.6 Females: 1.1	58.4%
LA (g)	14.7 (6.4)	Males: 14 Females: 11	57.7%
DHA + EPA (g)^a^	0.3 (0.4)	EFSA [58]: 0.25 ISSFAL [59]: 0.5 IOM [28]: 10% of LNA recommendation	43.1% 24.1% 59.1%
N-6/n-3 ratio	7.8 (2.6)		
PUFA (% of calories)	8.2 (2.7)	All: 6–11%	65.7%^b^
MUFA (% of calories)	12.1 (2.5)		
SFA (% of calories)	11.1 (2.9)	All: < 10%	35.0%
Total Fiber (g)	21.1 (9.3)	Males: 30 Females: 21	24.1%
Vitamin A (mcg)	1182.2 (788.6)	Males: 900 Females: 700	59.1%
Vitamin B6 (mg)	8.0 (18.7)	Males: 1.7 Females: 1.5	70.1%
Vitamin B12 (mcg)	16.1 (16.8)	All: 2.4	86.9%
Vitamin C (mg)	216.4 (280.0)	Males: 90 Females: 75	60.6%
Vitamin D (mcg)	32.4 (56.3)	51–70 yo: 15 Over 70: 20	40.9%
Vitamin E (mg)	8.8 (5.1)	All: 15	8.0%
Vitamin K (mcg)	167.9 (138.0)	Males: 120 Females: 90	55.5%
Calcium (mg)	1220.0 (603.3)	M 51–70 yo: 1000 Others: 1200	40.9%
Iron (mg)	17.9 (12.0)	All: 8	88.3%
Sodium (mg)	2933.0 (969.5)	51–70 yo: 1300 Over 70: 1200	91.2%
Potassium (mg)	2639.0 (744.1)	All: 4700	0.7%
Zinc (mg)	20.7 (17.9)	Males: 11 Females: 8	65.7%
Pantothenic acid (mg)	11.4 (12.8)	All: 5	62.8%
Choline (mg)	330.3 (120.4)	Males: 1.6 Females: 1.1	10.2%
Folate (mcg)	420.5 (203.8)	All: 400	41.6%
Water (g)	2105.5 (684.9)	Males: 3700 Females: 2700	10.2%
HEI	59.3 (15.6)		

Note *HEI* Health Eating Index, which can range from 0 to 100, *LNA* alpha-linolenic acid, *LA* linoleic acid, *PUFA* polyunsaturated fatty acids, *MUFA* monounsaturated fatty acids, *SFA* saturated fatty acids, *SD* standard deviation, *g* grams, *kcal* kilocalories, *FAO* Food and Agriculture Organization of the United Nations, *IOM* Institute of Medicine, *EFSA* European Food Safety Authority, *ISSFAL* International Society for the Study of Fatty Acids and Lipids

^a^There isn’t a clear consensus as to optimal intake of fatty acids other than LNA and LA. Recommendations for other fatty acids are typically based on total DHA and EPA

^b^Only 1.8% of children consumed more than 11% of their fat calories as PUFA, and 11.0% of older adults consumed more than 11.0% of their fat calories as PUFA

### Data reduction and analyses

SAS version 9.4 (SAS Institute, Cary, NC) was used for all analyses. Analysis of variance (ANOVA) with Tukey’s post-hoc tests were used to determine differences in fatty acid intake by categorical variables (e.g., education and age group) and dietary adequacy groups, and partial correlations and multivariable regressions were used to assess the relations between fatty acid intake, other nutrients, HEI scores, and continuous anthropometric variables (e.g., body mass index (BMI) and waist circumference). All analyses were run controlling for the contribution of total energy intake. Individuals who consumed more calories generally consumed more of every nutrient, and thus, partial correlations were calculated removing the shared variance explained by total energy.

Data were assessed for violations of assumptions of the general linear model, and none were found. Variables were fairly normally distributed without outliers, and there was no significant heterogeneity of variance in any models run. Intakes of many nutrients were highly correlated, and variance inflation factors (VIF) were checked for all multivariable regressions. VIFs offer a measure of how much the regression coefficients may be inflated due to collinearity among the included variables. VIFs above 10 are considered problematic in that the shared variance explained by the variables can make the coefficient estimates unreliable. No VIF above 10 were found.

## Results

### Children

The average n-6/n-3 ratio among children in these analyses was 9.2, ranging from 2.6 to 17.9. Most children consumed recommended amounts of LNA (71.8%) and LA (61.4%), but many fewer children consumed recommended amounts of DHA and EPA (6.8% - 14.1%). There is still some debate as to recommendations for fatty acids other than LNA and LA. DHA and EPA have been found to be important for health, particularly heart health, and recommendations have started to be made for a combination of DHA and EPA intake. In children, 200 mg was proposed by the Food and Agriculture Safety Organization of the United Nations (FAO) [35], and the Institute of Medicine (IOM) has recommended that DHA and EPA represent about 10% of recommended intake of LNA [28]. For children 7 to 12 years old, recommended LNA intake ranges from 0.9 g to 1.2 g, indicating the IOM recommends 0.09 g to 0.12 g (or 90 mg to 120 mg) intake of DHA and EPA. The IOM recommendations are quite a bit lower, and yet only 14.1% of children consumed recommended DHA and EPA for their age and gender.

The n-6/n-3 ratio, n-3 intake, and n-6 intake were not predicted by age, maternal education, infant mode of feeding, or BMI. No intakes of specific fatty acids were predicted by demographic or anthropometric variables. Among specific nutrients, lower n-6/n-3 ratios were associated with higher intake of almost all other nutrients (Table 3). The exception was vitamin E. Lower ratios were associated with lower intake of vitamin E (*r*(219) = 0.2, *p* < 0.05). In adequacy of intake analyses, children with lower ratios relative to those with higher ratios, controlling for energy intake, consumed adequate amounts of vitamin A (*F*(1,215) = 5.9, *p* < 0.05), vitamin D (*F*(1,215) = 8.4, *p* < 0.01), vitamin K (*F*(1,215) = 9.9, *p* < 0.01), phosphorus (*F*(1,215) = 4.2, *p* < 0.05), zinc (*F*(1,215) = 6.4, *p* < 0.01), pantothenic acid (*F*(1,215) = 8.3, *p* < 0.01), and water (*F*(215) = 9.5, *p* < 0.0001). There were only 9 children (4.1%) who had inadequate intake of vitamin B12, but those with adequate intake had significantly lower ratios (*F*(1,215) = 6.2, *p* < 0.05) than those without adequate intake. A multivariable regression was run including all nutrients for which adequacy of intake predicted the n-6/n-3 ratio, and vitamin D (*t*(209) = − 2.7, *p* < 0.05), vitamin K (*t*(209) = − 2.0, *p* < 0.05), and zinc (*t*(209) = − 2.1, *p* < 0.05) all predicted the ratio above and beyond the other nutrients and total energy.

**Table 3:** Correlations between fatty acid intake and other dietary nutrients partialling out the contribution of total energy

7- to 12-year-olds	n-3 intake	n-6 intake	HEI score	Fiber	Vit. B6	Vit. B12	Vit. C	Vit. D	Vit. E	Calcium	Potassium	Phosphorus	Pant. Acid	Niacin	Zinc	Choline
n-6/n-3 ratio	−0.59***	0.29***	−0.15*	0.05	−0.19*	− 0.22**	− 0.28***	− 0.37***	0.21**	− 0.29***	− 0.26**	− 0.3***	−0.29***	− 0.15*	−0.35***	− 0.35***
n-3 intake		0.48***	0.26**	−0.03	0.19**	0.23**	0.28***	0.28***	0.18*	−0.03	0.13	−0.01	0.24**	0.2**	0.12	0.14*
n-6 intake			0.12	−0.09	−0.05	−0.06	− 0.1	−0.23**	0.47***	−0.31***	− 0.3***	−0.28***	− 0.09	−0.01	− 0.26**	−0.24**
HEI score				0.6***	0.34***	0.26***	0.23**	0.38***	0.28***	0.35***	0.61***	0.48***	0.41***	0.28***	0.32***	0.43***
Fiber					0.29***	0.11	0.2**	0.2**	0.22**	0.23**	0.58***	0.37***	0.25**	0.23**	0.2**	0.26***
Vit. B6						0.78***	0.3***	0.43***	0.12	0.26***	0.21**	0.24**	0.82***	0.63***	0.41***	0.18*
Vit. B12							0.31***	0.41***	0.08	0.25**	0.2**	0.22**	0.78***	0.5***	0.31***	0.24**
Vit. C								0.57***	0.06	0.08	0.26**	0.09	0.39***	0.39***	0.22**	0.23**
Vit. D									0.05	0.47***	0.46***	0.47***	0.57***	0.51***	0.53***	0.42***
Vit. E										−0.01	0.07	0.02	0.17*	0.18*	0.14*	−0.04
Calcium											0.57***	0.83***	0.33***	0.07	0.44***	0.33***
Potassium												0.75***	0.35***	0.15*	0.36***	0.7***
Phosphorus													0.36***	0.15*	0.47***	0.61***
Pant. Acid														0.62***	0.61***	0.37***
Niacin															0.6***	0.24**
Zinc																0.41***
65- to 79-year-olds*	n-3 intake	n-6 intake	HEI score	Fiber	Vit. B6	Vit. B12	Vit. C	Vit. D	Vit. E	Calcium	Potassium	Phosphorus	Pant. Acid	Niacin	Zinc	Choline
n-6/n-3 ratio	−0.47***	0.35**	−0.21*	−0.002	0.03	−0.04	−0.22*	−0.22*	0.12	−0.33***	−0.3**	−0.18	−0.01	−0.24*	− 0.29*	−0.1
n-3 intake		0.61***	0.39***	0.07	0.19	0.17	0.23*	0.34**	0.1	0.17	0.08	0.12	0.12	0.32**	0.21*	0.02
n-6 intake			0.17	0.02	0.26*	0.12	0.02	0.11	0.17*	−0.13	−0.26*	−0.15	0.16	0.06	−0.09	− 0.06
HEI score				0.51***	0.19	0.18	0.24*	0.2*	0.37***	0.3**	0.67***	0.49***	0.21*	0.21*	0.31**	−0.03
Fiber					0.09	0.08	0.14	0.16	0.36**	0.27*	0.7***	0.4***	0.12	0.12	0.22*	−0.16
Vit. B6						0.69***	0.25*	0.21*	0.09	0.23*	0.11	0.13	0.83***	0.04	0.31**	−0.03
Vit. B12							0.24*	0.24*	0.13	0.39***	0.16	0.36**	0.79***	0.11	0.61***	−0.01
Vit. C								0.14	0.1	0.31**	0.21*	0.2*	0.25*	0.1	0.37***	−0.05
Vit. D									0.12	0.29**	0.11	0.3**	0.23*	0.44***	0.24*	0.08
Vit. E										0.19	0.23*	0.26*	0.23*	0.02	0.1	−0.21*
Calcium											0.43***	0.48***	0.42***	0.35**	0.53***	−0.05
Potassium												0.56***	0.18	0.11	0.34**	0.02
Phosphorus													0.28**	0.29**	0.38***	0.11
Pant. Acid														0.13	0.37***	−0.004
Niacin															0.07	0.02
Zinc																0.02

Note. *Partial correlations for older adults were calculated also partialling out the contribution of having a college degree. n-6/n-3 ratio: Omega-6 to omega-3 ratio; n-6: omega-6; n-3: omega-3; HEI: Health Eating Index; Vit.: Vitamin; Pant.: Pantothenic. *p < 0.05, **p < 0.01, ***p < 0.0001

The n-6/n-3 ratio was negatively correlated with HEI scores (*r*(218) = − 0.2, *p* < 0.05), and n-3 intake was positively correlated with HEI scores (*r*(219) = 0.3, *p* < 0.01) such that lower ratios and greater n-3 intake were associated with more healthful diets. Neither the n-6/n-3 ratio nor HEI scores were correlated with BMI (ratio: *r*(219) = 0.03, *p* > 0.05; HEI: *r*(219) = 0.01, *p* > 0.05). A multivariable regression was run to predict HEI scores with n-6 and n-3 intake, controlling for total energy. Greater n-3 intake predicted higher HEI scores (*t*(215) = 3.5, *p* < 0.0001), with each gram increase in n-3 intake predicting a 5.6 point increase in HEI score. Both consumption of LNA (*t*(215) = 2.6, *p* < 0.05) and the consumption of DHA + EPA (*t*(215) = 4.0, *p* < 0.05) predicted HEI scores with each gram increase in LNA predicting a 4.1 unit increase in HEI score and each gram increase in DHA + EPA predicting an 11.7 unit increase in HEI score. Neither n-6 intake nor total energy predicted HEI scores in children.

### Older adults

The average n-6/n-3 ratio among older adults was 7.8, and it ranged from 3.1 to 20.3. Just over half of the adults consumed at least the recommended amount of LNA (58.4%) and LA (57.7%). As with children, there is still debate as to the appropriate recommendations for other fatty acids. More adults met all of the recommendations for DHA and EPA, with 59.1% meeting the IOM standard of 10% of recommended LNA, 43.1% reaching the European Food Safety Authority (EFSA) recommendation of 250 mg, and 24.1% reaching the International Society for the Study of Fatty Acids and Lipids (ISSFAL) recommendation of 500 mg.

Older adults with at least a college degree consumed a significantly lower ratio than those without a college degree, *F*(1,125) = 4.3, *p* < 0.05. Older adults with a college degree consumed a ratio one unit lower than the ratio consumed by adults without a college degree (8.1 vs 7.2). However, there weren’t any significant differences in consumption of total n-6 or total n-3 among older adults. Older adults with a college degree consumed significantly more arachidonic acid (ARA, n-6, *F*(1,125) = 5.4, *p* < 0.05) and docosapentaenoic acid (DPA, n-3, *F*(1,125) = 6.1, *p* < 0.05). Older adults with a college degree also consumed a healthier diet in general based on the HEI scores, *F*(1,125) = 10.2, *p* < 0.01. Those with a college degree had an average HEI score of 64.9 compared to 56.2 for those without a college degree. Similar to the 7- to 12-year-olds, lower n-6/n-3 ratios were related to higher intake of several other nutrients (Table 3). Lower ratios were also associated with lower sodium intake (*r*(133) = 0.3, *p* < 0.01). Consuming more total n-3 was correlated with a smaller waist circumference (*r*(126) = − 0.2, *p* < 0.05), and lower diastolic blood pressure was also associated with consuming more DHA (*r*(126) = − 0.2, *p* < 0.05) and EPA (*r*(126) = − 0.2, *p* < 0.05).

Older adults with adequate intake of vitamin D (*F*(1,130) = 16.0, *p* < 0.01), calcium (*F*(1,130) = 7.5, *p* < 0.05), and water (*F*(1,130) = 3.9, *p* < 0.05) had significantly lower ratios than those who did not have adequate intake. There were few adults with adequate intake of vitamin E (*n* = 11, 8.0%). However, older adults with adequate vitamin E intake had higher ratios than those without adequate vitamin E intake (*F*(1,130) = 5.8, *p* < 0.05). A multivariable regression was run including all nutrients for which adequacy of intake predicted the n-6/n-3 ratio, and vitamin E (*t*(121) = 2.4, *p* < 0.05) and calcium (*t*(121) = − 3.9, *p* < 0.01) predicted the ratio above and beyond the other nutrients, total energy, and having a college degree.

HEI scores were correlated with the n-6/n-3 ratio (*r*(133) = − 0.3, *p* < 0.01), n-3 intake (r(133) = 0.4, p < 0.01), and n-6 intake (r(133) = 0.2, *p* < 005). A multivariable regression was run to determine the relation between n-3 and n-6 intake and overall healthy eating, controlling for total energy and having a college degree. Greater n-3 intake predicted higher HEI scores (*t*(123) = 4.3, *p* < 0.0001) with every one gram increase in n-3 predicting a 9.7 point increase in HEI score. Consuming more kilocalories predicted a decrease in HEI scores (*t*(123) = − 4.9, *p* < 0.0001) with every kilocalorie increase in total energy predicting a 0.01 point decrease in HEI score. Both LNA intake (*t*(123) = 3.2, *p* < 0.05) and DHA + EPA intake (*t*(123) = 5.8, *p* < 0.05) predicted HEI scores with every gram increase in LNA predicting a 5.6 unit increase in HEI scores and every gram increase in DHA + EPA predicting a 17.9 unit increase in HEI. n-6 intake was not related to overall healthy eating. More healthy eating was also associated with lower BMI (*r*(126) = − 0.3, *p* < 0.05) and smaller waist circumference (*r*(126) = − 0.3, *p* < 0.05).

## Discussion

Results from these analyses indicated that children and older adults generally consumed more LNA than recommended and approximately the recommended amount of LA, whereas only a small percentage consumed the recommended amounts of the longer-chain fatty acids, EPA and DHA. In all, 71.8% of 7- to 12-year-olds and 58.4% of 65- to 79-year-olds consumed at least the recommended amount of LNA for their age and gender. Regardless of the recommendation used, children 7 to 12 years of age rarely consumed recommended amounts of DHA and EPA (up to 14.1% reached recommendations). Adults were more likely to reach recommended DHA and EPA intake (at least 24.1% reached recommendations), but the differing recommendations produced more variability with almost 60% of adults reaching IOM recommendations. The n-6/n-3 ratios were slightly lower (9.2 in children and 7.8 in older adults) than would be produced by consuming recommended quantities of LA and LNA. The n-6/n-3 ratios were as low as 2 (children) or 3 (adults) and as high as 17 (children) or 20 (adults). No n-6/n-3 ratios above 20 were found in children or older adults.

It is not surprising that the intake of EPA and DHA across the ages does not meet the recommended amounts. The standard American diet has long been criticized for its low EPA and DHA content [2, 3, 36]. Even health conscious Americans have difficulty meeting their needs given the paucity of, and therefore prohibitive cost of, quality grass-fed meats and wild-caught fatty fish, which are the best sources of the longer chain omega-3. It is further thought that the metabolic pathway through which DHA is synthesized from LNA is not efficient [37, 38]. Certainly, the n-6 and n-3 pathways, which share a finite, genetically-controlled supply of desaturases and elongases, are only efficient if the intake of LA and LNA are in balance. Thus, it has been suggested [39] that the best way to increase omega-3 availability in the body is to decrease omega-6 intake. We have shown previously that the n-6/n-3 ratio is very important for brain health, especially when qualified by omega-6 and omega-3 intakes [9, 29]. It is possible that many of the disease states prevalent in the United States – attention deficit hyperactive disorder, depression, cardiovascular disease, metabolic syndrome, mild cognitive decline (to name only a few) – could be ameliorated by increasing EPA and DHA intake. Additionally, as the present data indicate, overall healthier diets result in a more reasonable balance between the two competing fatty acid metabolic pathways, which may lead to a metabolic increase in the availability of DHA and EPA.

Specifically, as measured by HEI scores and the ability to meet individual nutrient recommendations, healthier diet consumption was related to lower n-6/n-3 ratios in children from the present sample. Lower n-6/n-3 ratios were associated with higher intake of almost every other nutrient. On the surface, these results would imply that households with a better understanding of and ability to meet nutrient requirements are consuming healthier diets across the board and likely increasing n-3 intake or keeping n-6 intake in check. However, no demographic variables tested (which included maternal education, BMI, and infant mode of feeding) were related to n-6/n-3 ratios in children. It could be that recommendations for the n-6/n-3 ratio are helpful for improving overall diet for a variety of households. Promoting foods high in n-3 fatty acids, such as fatty fish, grass-fed meats, flaxseed, chia seeds, soybeans, and spinach, may promote other healthy dietary habits. For instance, spinach is a common green used in salads, and consuming more salads may also increase consumption of other vegetables. Exposure to foods has been linked to increased food acceptance and consumption among children [40–42]. The n-6 and n-3 fatty acids are frequently consumed through cooking oils [2], and it could also be that messages about increasing n-3 have prompted families to switch from high n-6 oils to more moderate n-6 oils or even high n-3 oils. Because oils high in n-3 cannot be used for frying due to low smoke points, moving from high n-6 oils to n-3 oils may also mean a reduction in the consumption of fried foods that are frequently linked to less healthy dietary patterns and poor health outcomes [43–46].

For older adults, consuming a healthier diet was also related to having lower n-6/n-3 ratios, but lower n-6/n-3 ratios were associated with higher intake of fewer nutrients. Only having a college degree as an older adult predicted consuming a diet with a lower n-6/n-3 ratio. n-3 intake was related to waist circumference, and DHA and EPA intake were related to diastolic blood pressure. Recommendations around n-3 fatty acids have only become part of the mainstream conversation in recent decades. Older adults may have established their dietary habits well before recommendations around n-3 fatty acids and DHA became prominent. Those with a college education may have access to better nutrition information or find it easier to adapt to new recommendations than those without a college education. The older adults in this sample also frequently used n-3 supplements (43.6%, *n* = 58), and they may have reduced their n-6/n-3 ratios through n-3 supplements without making any changes to the rest of their diet. The children in this sample infrequently consumed n-3 supplements (6.9%, *n* = 15), and changes to the n-6/n-3 ratio may have been more closely linked to changes in overall dietary patterns.

Despite changes in n-3 availability in the food supply [2, 13, 47] that have caused many researchers to be concerned about the effect of n-3 consumption and the overall balance of n-6 and n-3 in a typical diet, most children and older adults in the present analyses were able to consume recommended amounts of LNA and not significantly exceed recommendations for LA. Participants were less likely to consume recommended amounts of DHA and EPA, but that issue did not result in significantly elevated ratios. There is some evidence that the overall balance of n-6 and n-3 fatty acids is not helpful when considering heart health [48], but the inclusion of the n-6/n-3 ratio has been fruitful in investigations of cognitive function [29, 49–51]. These differences could reflect a fundamental tenet of nutrition research that the “tissue is the issue” [52] as our diets support a variety of tissues with a variety of functions. The role of each nutrient can vary within each tissue, and the overall recommendations for consumption will need to reflect the varied roles each nutrient can play. However, differences in the role of the n-6/n-3 ratio in heart health and cognitive function could also reflect the need to better clarify the relation between total intake of n-6 and n-3 fatty acids and their overall balance. Consideration of one family of fatty acids without the other ignores the importance of both elements and obfuscates the role of fatty acids in human health.

Additionally, it is likely that there is more than one optimal pattern of n-6 and n-3 intake. Considering genetics [23, 24], developmental period [51], and social environment will aid practitioners in making the best recommendations for a given individual. For instance, we previously reported an interaction between n-3 intake and the n-6/n-3 ratio such that consumption of higher amounts of n-3 and very high amounts of n-6 (resulting in a higher ratio) and consumption of lower amounts of n-3 and very low amounts of n-6 (resulting in a lower ratio) were both predictive of better performance on planning tasks in children [29, 51]. Children were able to perform well with low or high n-3 intake, if their overall balance of n-6 and n-3 fatty acids was appropriate. It is possible to support cognitive function in a variety of environments, even those with minimal access to sources of n-3 fatty acids.

One issue that needs to be addressed is that current recommendations do not match the research, and the research does not match observed dietary patterns. Researchers have remained consistent in the recommendation that ratios are more beneficial when lower, with 4:1 posited as a potential goal [52, 53]. These findings may result from the lower n-3 intake common in the United States, but that low intake is an important consideration. Current recommendations have people consuming ratios between 7:1 and 11:1, which does not match findings that lower ratios are better, especially if there is low underlying n-3 intake as is so commom in the United States and other countries such as Australia. Recommendations should be adjusted to more closely match evidence reported in the literature. Researchers should also take into account the underlying balance of n-6 and n-3 fatty acids when conducting supplementation studies. In both animals and humans, there has been limited consideration of the n-6/n-3 ratio when conducting randomized trials, which presents a potential confound. Animal studies frequently create extremely high ratios (above 100:1) with deficiency models that do not reflect observed diets [5, 8, 54]. Human supplementation studies either do not consider the baseline balance of n-6/n-3 fatty acids (therefore obscuring how that changes with supplementation) or utilize placebo oils that can inflate the n-6/n-3 ratio in the control participants [55–57]. Both issues could and should be addressed to provide even better recommendations for n-6 and n-3 intake.

The presented analyses should be interpreted in light of a few limitations. First, the children and older adults were all from a single region of the United States, and therefore do not represent all American diets. The overall nutrient intake values found were not considerably different from those reported in nationally representative datasets like NHANES, but further work replicating these results should be conducted in other regions. These data were not collected from a coastal city or other specific location where increased access to fish (a source of large amounts of n-3 FAs) could potentially skew results, but location may very well be a relevant factor for intake of foods that affect n-6 and n-3 fatty acids. Recruitment from a single region also reduced the ethnic and racial diversity of the sample, and additional studies will be conducted with more diverse samples. Additionally, dietary intake was estimated using 24-h diet recalls. These recalls were conducted using standardized and validated protocols designed to collect the most accurate diet data, but highly accurate dietary intake data are notoriously difficult to collect. Any study presenting dietary intake data needs to be replicated to increase confidence in the results. Additionally, future studies will include biomarkers of fatty acid status and fatty acid metabolism genes to further increase confidence in results.

## Conclusion

In conclusion, individuals in the Southeastern United States are able to consume recommended amounts of the shorter chain, but not the longer chain n-6 and n-3 fatty acids. Recommendations for DHA and EPA can be improved with studies that better reflect current dietary patterns, by including genetic and epigenetic mechanisms that affect fatty acid metabolism, and most importantly, by considering both n-6 and n-3 intake and their balance. Recommendations for n-6 and n-3 intake and their ratio may also be a way to promote overall healthier diets, especially in children. Nutritional needs change with development, and each of the above factors needs to be considered at different points in time. Better recommendations should lead to improved outcomes as people have managed to follow the clear recommendations offered for LNA and LA intake, regardless of changes to the food supply.

## Abbreviations

AI: Adequate intake
ANOVA: Analysis of variance
BMI: Body mass index
DHA: Docosahexaenoic acid
EFSA: the European Food Safety Authority
EPA: Eicosapentaenoic acid
FAO: Food and Agriculture Safety Organization of the United Nations
HEI: Healthy Eating Index
IOM: Institute of Medicine
ISSFAL: International Society for the Study of Fatty Acids and Lipids
LA: Linoleic acid
LNA: alpha-linolenic acid
MoCA: Montreal Cognitive Assessment
n-3: omega-3
n-6: omega-6
NDSR: Nutrition Data System for Research
NHANES: National Health and Nutrition Examination Survey
RDA: Recommended daily allowances
SNPs: Single nucleotide polymorphisms
VIF: Variance inflation factors
